# 

**Published:** 1852-05

**Authors:** 


### NOTICE TO CORRESPONDENTS.

Communications and Books for notice should be addressed to the Editors, care of Messrs. Lindsay & Blakiston.

Letters, &c., connected with the *business affairs* of the Journal should be addressed to the Publishers.

Papers for publication must be received *before* the 20th of the month, or they cannot appear in the forthcoming number.

The following Journals have been received in exchange:

New York Medical Times.
Medical News. Philadelphia.
American Journal of Medical Sciences. April.
Buffalo Journal.
L'Union Medicale.
New York Medical Gazette.
The Boston Medical and Surgical Journal. (Weekly.)
Stethoscope and Virginia Medical Gazette.
Western Journal.
Buffalo Journal.
Transylvania Medical Journal.
Nashville Journal of Medicine and Surgery.
Southern Medical and Surgical Journal.
New Hampshire Journal of Medicine.
Opal, from the Utica Insane Asylum.
Dental Times and Advertiser.
The London Lancet. (Weekly, London.)
The Medical Times and Gazette. (Weekly, London.)
Dublin Medical Press.
London Journal of Medicine.
British and Foreign Medico-Chirurgical Review.
Edinburgh Monthly Journal.
Journal of Psychological Medicine.
Journal of Pharmacy.
North Western Medical Intelligencer.
American Journal of Insanity.

---

### BOOKS RECEIVED.

Report of the Ohio Lunatic Asylum for the year 1851.
Swett on Diseases of the Chest. From the Publishers.
Graham's Elements of Chemistry. From Blanchard & Lea.
Miller's Principles of Surgery.
Mendenhall's Vade Mecum.
Theses. From E. Brown-Séquard, M. D.

---

Communications have been received from:—

E. B. Gardette, M. D., Philadelphia.
Lewis Slusser, M. D., Canal Fulton, Ohio.
E. D. Daily, M. D., Maryland.
D. H. Gibson, M. D., Choctaw Nation.
T. M. Jacks, Arkansas.

---

The foreign correspondents of the Examiner will please direct their Exchanges and other communications to the care of Mr. Thos. Delf, No. 12 Paternoster Row, London, or Mr. H. Bossange, 21 Bis, Quai Voltaire, Paris.

